# OTUB1-mediated inhibition of ubiquitination: a growing list of effectors, multiplex mechanisms, and versatile functions

**DOI:** 10.3389/fmolb.2023.1261273

**Published:** 2024-01-09

**Authors:** Miaomiao Wu, Lidong Sun, Tanjing Song

**Affiliations:** ^1^ Deparment of Obstetrics and Gynecology, Shuyang Hospital of Traditional Chinese Medicine, Suqian, China; ^2^ Department of Biochemistry and Molecular Biology, School of Basic Medicine, Tongji Medical College, Huazhong University of Science and Technology, Wuhan, Hubei, China; ^3^ Cell Architecture Research Institute, Huazhong University of Science and Technology, Wuhan, Hubei, China

**Keywords:** OTUB1, ubiquitination, deubiquitinase, degradation, cancer, ubiquitin-conjugating enzyme

## Abstract

Protein ubiquitination plays a pivotal role in protein homeostasis. Ubiquitination may regulate the stability, activity, protein–protein interaction, and localization of a protein. Ubiquitination is subject to regulation by two groups of counteracting enzymes, the E3 ubiquitin ligases and deubiquitinases. Consistently, deubiquitinases are involved in essentially all biological processes. OTUB1, an OTU-family deubiquitinase, is a critical regulator of development, cancer, DNA damage response, and immune response. OTUB1 antagonizes the ubiquitination of a wide-spectrum of proteins through at least two different mechanisms. Besides direct deubiquitination, OTUB1 can also inhibit ubiquitination by non-canonically blocking ubiquitin transfer from certain ubiquitin-conjugases (E2). In this review, we start with a general background of protein ubiquitination and deubiquitination. Next, we introduce the basic characteristics of OTUB1 and then elaborate on the updated biological functions of OTUB1. Afterwards, we discuss potential mechanisms underlying the versatility and specificity of OTUB1 functions. In the end, we discuss the perspective that OTUB1 can be a potential therapeutic target for cancer.

## 1 Introduction to ubiquitination

Protein carries out most biological functions. Protein homeostasis is necessary for cellular function and its dysregulation causes various human diseases, including cancer and immune dysfunction. The expression, localization, protein–protein interaction, and activity of a protein are all subject to exquisite regulation. Protein ubiquitination plays a key role in all these processes. Protein degradation is the prototypical function of ubiquitination. Protein degradation, just like protein biosynthesis, represents a major mechanism for protein homeostasis. Protein degradation not only removes damaged protein and recycles amino acids but also removes no more needed proteins to allow critical cellular processes, which, for example, have been well proven for cell cycle progression (Dang et al., 2021). Besides protein degradation, it is now well-appreciated that ubiquitination has various other functions. Ubiquitination can regulate protein localization, protein–protein interaction, and protein activity. The contextual consequences of ubiquitination have to do with the topologies of the ubiquitin chain. Ubiquitination can occur in the form of monoubiquitination or polyubiquitination. Polyubiquitination can occur in different "topologies”. As ubiquitin contains seven lysines (6, 11, 27, 29, 33, 48, and 63), there are at least eight different topologies for ubiquitin–ubiquitin linkage, with the eighth one being the linear, i.e., Met1-linked. While the K48-linked ubiquitin chain is most notably connected with degradation, other topologies like the K63-linked ubiquitin chain can function in other aspects (Komander and Rape, 2012; Yau and Rape, 2016).

**FIGURE 1:**
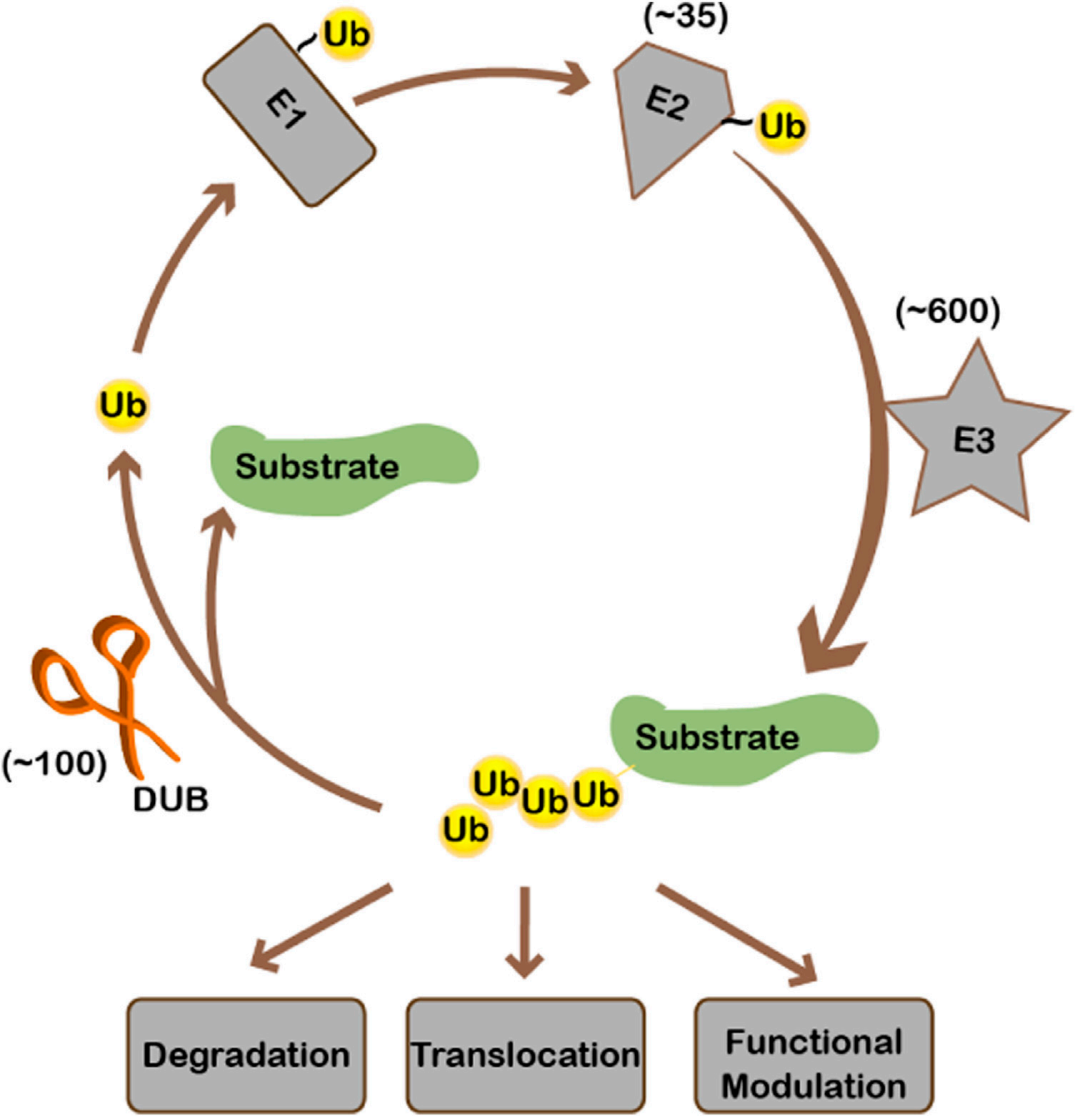
Ubiquitination and deubiquitination.

Ubiquitination, like many other important biological events, is a highly-regulated enzyme-catalyzed process. Ubiquitin is first activated by and linked to E1 to form a thioester bond. Then ubiquitin is transferred to E2, the ubiquitin conjugating enzyme. Finally, E3 ubiquitin ligases transfer ubiquitin from E2 to substrates. There is one E1, 35 E2s, and hundreds of E3s for ubiquitin in human cells. Substrate specificity is mainly realized by E3 ubiquitin ligases (Figure 1). On top of that, when, where, and to what extent a substrate gets ubiquitinated is regulated by environmental stimuli. Per signaling cues, ubiquitination is dynamically regulated. Ubiquitination can be removed from the substrate by deubiquitinases (DUBs) (Figure 1). About 100 proteins have been identified or predicted to be deubiquitinases in the human genome since the discovery of the UCH (ubiquitin C-terminal hydrolase) and USP (ubiquitin-specific protease) families of deubiquitinases in the 1980s and early 1990s, respectively (Wilkinson et al., 1989; Tobias and Varshavsky, 1991). DUBs fall into seven families according to the catalytic domains, including the USP, UCH, JAMM, OTU, MJD, MINDY, and ZUP1 families (Mevissen and Komander, 2017). Except for the JAMM family being metalloproteases, all other families are cysteine proteases. For the OTU-domain family, there are 16 active DUBs in human (Keusekotten et al., 2013), which can be further categorized into four subfamilies: the OTU domain-containing ubiquitin aldehyde-binding proteins (Otubains) subfamily (OTUB1 and OTUB2), the OTU domain-containing proteins (OTUDs) subfamily (OTUD1, OTUD2/YOD1, OTUD3, OTUD4, OTUD5/DUBA, OTUD6A, OTUD6B, and ALG13), the A20-like subfamily (A20, Cezanne/OTUD7B, Cezanne2/OTUD7A, TRABID/ZRANB1, and VCPIP), and OTULIN with linear linkage specificity (Keusekotten et al., 2013).

Ubiquitination starts from the activation of ubiquitin by E1. Ubiquitin is then conjugated to E2, after which ubiquitin is transferred to the substrate by E3 ligase. Ubiquitination may cause substrate degradation or alternatively change the localization/activity of the substrate. Ubiquitinated substrate can be deubiquitinated by deubiquitinases, during which ubiquitin is recycled. There is approximately 1 E1, 35 E2s, 600 E3s, and 100 deubiquitinases encoded in the human genome.

## 2 Introduction to OTUB1

**FIGURE 2:**
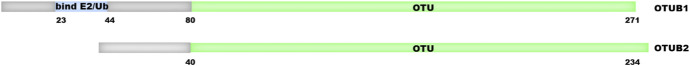
Comparison between domain arrangement of OTUB1 and OTUB2.

OTUB1 is the founding member of OTU-domain deubiquitinases. It is expressed abundantly in the kidney, spleen, prostate, brain, liver, lung, and testis (Balakirev et al., 2003). Upon its discovery, OTUB1 was shown to possess iso-peptidase activity towards polyubiquitin chains. The structure and biochemical activity of OTUB1 have both similarities and uniqueness compared to other deubiquitinases. Biochemical analysis reveals that OTUB1 has a strong preference for Lys48-linked polyubiquitin chains over other topologies (Edelmann et al., 2009; Mevissen et al., 2013). Besides ubiquitin, OTUB1 is capable of cleaving other ubiquitin-like modifiers, including NEDD8 (neural-precursor-cell-expressed developmentally downregulated 8) but not SUMO (small ubiquitin-related modifier) 1/2/3 or ISG15 (interferon-stimulated gene 15) conjugates (Edelmann et al., 2009). As with most other DUBs, OTUB1 is a cysteine protease. Cysteine-91, histidine-265, and aspartate-267 comprise the catalytic triad directly responsible for catalysis. Within the OTU family DUBs, OTUB1 shares the highest homology with OTUB2 (Zhu et al., 2021b). However, the pocket accommodating p1’ (the residue C-terminus to the cleaved bond) is narrower in OTUB1 than in OTUB2. Additionally, OTUB1 contains an extra N-terminal alpha-helix which contacts the proximal ubiquitin (Figure 2) (Messick et al., 2008; Edelmann et al., 2009; Mevissen et al., 2013; Altun et al., 2015). These observations may explain why OTUB1 preferentially cleaves the K48-linked ubiquitin chain and cannot cleave ubiquitin fusion proteins (Messick et al., 2008; Edelmann et al., 2009; Mevissen et al., 2013; Altun et al., 2015). Importantly, the N-terminal alpha-helix can also contact and inhibit E2 (Figure 1), which, as elaborated below, represents a major mechanism for OTUB1 function.

Both OTUB1 and OTUB2 have OTU domain in the c-terminus, which has catalytic activity. In addition, they have an N-terminal extension. Compared to OTUB2, OTUB1 has a longer N-terminal extension, which is critical for binding to E2 or E2∼Ubiquitin.

Studies have shown the important roles of OTUB1 in development, physiological homeostasis, and various diseases (Saldana et al., 2019; Zhu et al., 2021b; Liao et al., 2022). Notably, multiple pieces of evidence show OTUB1 functions in cancer, DNA damage repair, and immunity. Mechanistically, OTUB1 antagonizes the ubiquitination of different proteins, which may change their stability or activity. With increasing interest in OTUB1 function, OTUB1 has been the topic of a few elegant reviews (Saldana et al., 2019; Liao et al., 2022). Since their publication, plenty of important findings have been newly made on OTUB1. For example, dozens of publications on OTUB1 have been published in 2023 alone, which will be discussed below. When discussing OTUB1 functions, we mainly focus on the cases where the substrates or direct effectors of OTUB1 have been identified. In addition, we discuss the common themes underlying OTUB1 function, including how OTUB1 represses ubiquitination and how OTUB1 is regulated. In the end, we discuss the possibility of OTUB1 as a therapeutic target in cancer.

## 3 Common paradigm of OTUB1 function

**FIGURE 3:**
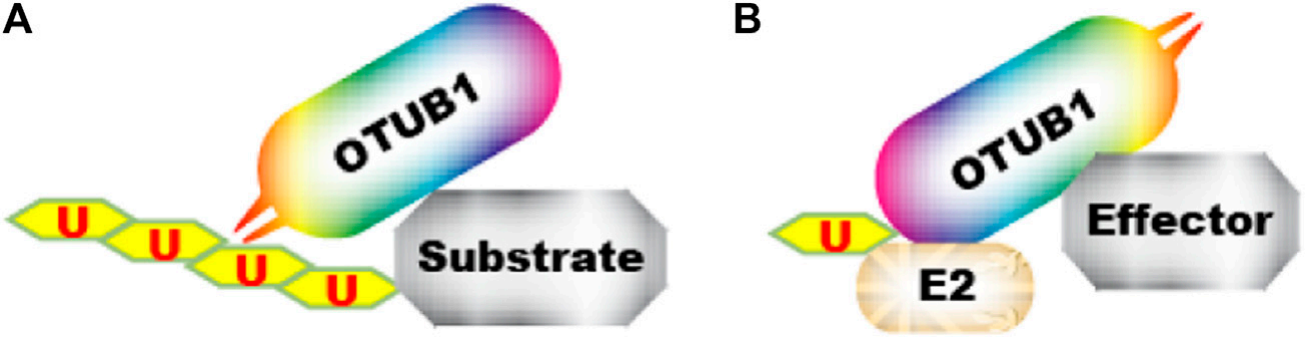
Two mechanisms by which OTUB1 antagonizes ubiquitination. **(A)** OTUB1 interacts with a ubiquitinated protein and removes ubiquitin from it. **(B)** OTUB1 interacts with E2∼Ub conjugate and locks it in an inactive conformation, preventing ubiquitin transfer to an “effector”. 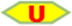
denotes ubiquitin.

OTUB1 functions through multiple downstream effectors. The first type of effectors are direct substrates of OTUB1’s deubiquitinase (DUB) activity (Figure 3A). In addition, OTUB1 can also inhibit protein ubiquitination through another mechanism. In the latter case, OTUB1 binds ubiquitin-conjugated E2 and blocks it in an inactive state to prevent ubiquitin transfer. OTUB1 engages E2 with an N-terminal alpha-helical extension which is absent in OTUB2 (Nakada et al., 2010; Juang et al., 2012; Wiener et al., 2012) (Figure 2). Most OTUB1 effectors identified so far fall into the latter category since the initial discovery in 2010 (Nakada et al., 2010) (Figure 3B). Effectors in both categories generally interact with OTUB1 so even for proteins in the second category, the specificity is warranted. On top of that, the effect of OTUB1 on an effector is regulated spatially and temporally.

### 3.1 OTUB1 directly deubiquitinates some substrates

A few direct substrates of OTUB1 have been identified, several of which are transcription factors (Table 1). Consistent with the ubiquitin chain topology preference of OTUB1, the ubiquitin chains on these substrates are also K48-linked. OTUB1 functions to stabilize most of these proteins except ERα, which is consistent with the prototypical role of the K48-linked ubiquitin chain in protein degradation (Table 1).

**TABLE 1:** Direct substrates of OTUB1.

Substrate	Effect	References
ERα	Decrease in activity	Stanisic et al. (2009)
FOXM1	Protein stabilization	Karunarathna et al. (2016)
		Wang et al. (2016)
SNAIL	Protein stabilization	Zhou et al. (2018)
UBC13	Protein stabilization	Mulas et al. (2021)
PD-L1	Protein stabilization	Zhu et al. (2021a)
c-MYC	Protein stabilization	Han et al. (2022)

The direct substrates of OTUB1 (the first column), the biological effects of their deubiquitination by OTUB1 (the second column), and corresponding references (the third column) are listed.

### 3.2 OTUB1 represses ubiquitination through the non-canonical mechanism

While a few proteins are direct substrates of OTUB1’s DUB activity, more proteins are regulated by OTUB1 through the non-canonical mechanism (Table 2). To determine whether OTUB1’s DUB activity is involved, a typical experiment is to examine the effects of catalytic inactive mutants of OTUB1, most often C91A or C91S. On the other hand, when catalytically dead mutants are still effective toward a protein, to disrupt the non-canonical function of OTUB1, a few different mutants have been used in the past. One mutant is D88A. Anecdotally, D88 was initially proposed to be part of the catalytic triad based on its consensus in sequence alignment (Nakada et al., 2010; Wu et al., 2021). But later structural studies show aspartate in catalytic triad should be D267 instead of D88 (Edelmann et al., 2009; Juang et al., 2012; Wiener et al., 2012). D88A disrupts the interaction between OTUB1 and E2 and hence the non-canonical function of OTUB1 in most scenarios (Nakada et al., 2010; Sun et al., 2012). Another mutant used to disrupt OTUB1’s non-canonical function is the N-terminal deletion mutant (ΔN), which lacks the N-terminal alpha-helix consisting of about 45 amino acids (Nakada et al., 2010). This N-terminal alpha-helix engages ubiquitin-conjugated E2 as shown by structural study (Juang et al., 2012; Wiener et al., 2012).

**TABLE 2:** Proteins regulated by OTUB1 through the non-canonical mechanism.

Effector	Effect	References
DNA damage foci	DNA damage repair	Nakada et al. (2010)
P53	Stabilization	Sun et al. (2012)
Smad3	Stabilization	Herhaus et al. (2013)
RAS	Change in localization	Baietti et al. (2016)
MDMX	Stabilization	Chen et al. (2017)
UBE2E1	Stabilization	Pasupala et al. (2018)
SLC7A11	Stabilization	Liu et al. (2019)
SOCS1	Stabilization	Wang et al. (2019b)
AKT	Decrease in activity	Zhou et al. (2019a)
P100	Stabilization	Li et al. (2019)
Nur77	Stabilization	Pei et al. (2019)
RIG-I	Stabilization	Jahan et al. (2020)
MSH2	Stabilization	Wu et al. (2021)
DEPTOR	Stabilization	Zhao et al. (2018)
c-Maf	Stabilization	Xu et al. (2021)
HIF1α	Stabilization	Liu et al. (2022)
YAP1	Stabilization	Yan et al. (2022)
CCN6	Stabilization	Zhao et al. (2023)
FGFR2	Stabilization	Zhu et al. (2023)

The effectors of OTUB1 via the non-canonical mechanism (the first column), the biological effects of their deubiquitination by OTUB1 (the second column), and corresponding references (the third column) are listed.

### 3.3 Other mechanisms by which OTUB1 regulates ubiquitination

Besides the two major mechanisms elaborated above, OTUB1 can regulate protein ubiquitination through other mechanisms. For example, OTUB1 bridges interaction between GRAIL and deubiquitinase USP8, thus promoting the deubiquitination of GRAIL (Soares et al., 2004). In addition, there are other proteins reported to be regulated by OTUB1, albeit the exact mechanisms require further delineation. These proteins include TRAF3 (Li et al., 2010; Peng et al., 2014; Xie et al., 2020), TRAF6 (Li et al., 2010; Zhang et al., 2022), YB-1 (Dong et al., 2015), RPA1 (Wang et al., 2015), Cyclin E1 (Liao et al., 2020), ATF6 (Zhang et al., 2021a), E2F1 (Zhang et al., 2021b), IAP-1 (Koschel et al., 2021), EYA1 (Xie et al., 2021), HSF1 (Ling et al., 2022), IRF7 (Xing et al., 2023), TAZ (Nakagawa et al., 2023), and RACK1 (Li et al., 2023b).

### 3.4 Mechanisms underlying substrate specificity of OTUB1

More and more effectors of OTUB1 are being identified. One question that has emerged is how OTUB1 can regulate these effectors with specificity, especially considering at least seven (UBE2N, UBE2D1-3, and UBE2E1-3) out of all 35 E2s are inhibited by OTUB1. Current evidence suggest one major mechanism underlying OTUB1’s specificity is protein–protein interaction. As discussed above, the interaction with OTUB1 is detected for almost all OTUB1 effectors, presumably in a dynamic fashion. These findings hint that the inhibitory effect of OTUB1 on E2 is not equally distributed among all proteins in the cell, but rather preferentially directed towards those interacting with OTUB1. OTUB1’s interaction with others, activity, localization, and expression are also delicately regulated. Firstly, multiple mechanisms regulate the protein–protein interaction between OTUB1 and its effectors. OTUB1 is subject to post-translational modifications, including lysine-methylation, mono-ubiquitination, phosphorylation, and S-glutathionylation, which either promote or suppress the effect of OTUB1 on a certain protein (Li et al., 2014; Aboushousha et al., 2023; Deng et al., 2023; Seo et al., 2023) (Table 3). Secondly, interaction between OTUB1 and effectors can be facilitated by other scaffold-like proteins (Peng et al., 2014; Liu et al., 2019; Li et al., 2023a; Zhang et al., 2023). Thirdly, interaction involving OTUB1 can be regulated by post-translational modifications (PTM) of OTUB1 interaction partners. For example, ERK/RSK-mediated phosphorylation of Y-box binding protein-1 aggravates diabetic cardiomyopathy by suppressing its interaction with deubiquitinase OTUB1 (Zhong et al., 2022). Fourthly, the DUB activity of OTUB1 can be regulated as well. Post-translational modifications including FAT10, hydroxylation, persulfidation, and ubiquitination on OTUB1 are reported to regulate its DUB activity (Scholz et al., 2016; Bialas et al., 2019; Pickel et al., 2019; Chen et al., 2021; Kim et al., 2021) (Table 3). In addition, interaction with uncharged E2s can promote the DUB activity of OTUB1 (Wiener et al., 2013). Fifthly, OTUB1 interacts with its effectors in different subcellular compartments. OTUB1 exhibits very different subcellular localization in different cell types or under different stimuli. Subcellular localization of OTUB1 is regulated by multiple factors (Table 3). For example, Ser-16 phosphorylation by casein kinase 2 triggers OTUB1 nuclear localization (Herhaus et al., 2015). In contrast, IL-15 induces the partial redistribution of OTUB1 to the plasma membrane (Zhou et al., 2019a) while virus infection causes OTUB1 to be mainly distributed to mitochondria (Jahan et al., 2020). In conclusion, the temporal regulation of OTUB1’s localization, activity, and interaction with others can render specificity towards different effectors.

**TABLE 3:** Post-translational modifications of OTUB1.

Site	Modification	Function
S16	Phosphorylation	Promote nuclear localization Zhu et al. (2021b)
Y26	Phosphorylation	Promote OTUB1-RAPTOR interaction Seo et al. (2023)
C23, C91	Nitrosylation	Inhibit activity Kumari et al. (2022)
N22	Hydroxylation	Change interactome (Scholz et al. (2016)
C91	Persulfidation	Increase interaction with SLC7A11 Chen et al. (2021)
K122	Methylation	Inhibit interaction with UBC13 (Deng et al., 2023)
C23, C204	S-glutathionylation	Promote interaction with E2 Aboushousha et al. (2023)
uncharacterized	FAT10	Proteasomal degradation Bialas et al. (2019)
K59, K109	Monoubiquitination	Promote E2-inhibiting activity Li et al. (2014)

The modified sites (the first column), the types of modification (the second column), and the biological effects of these modifications together with corresponding references (the third column) are listed.

## 4 OTUB1 has versatile functions

### 4.1 OTUB1 in development

Studies on mouse models have shown the importance of OTUB1 in the development of lung and bone. Ruiz-Serrano et al. found global OTUB1 knockout causes perinatal lethality by asphyxiation (Ruiz-Serrano et al., 2021), which is due to defective lung development. Mechanistically, the loss of OTUB1 increases mTOR activity in lung cells, which increases cell proliferation, decreases saccular air space, and prevents inhalation (Ruiz-Serrano et al., 2021). Zhu et al. found whole-body OTUB1 deletion in mice leads to shorter body length, which is due to defective bone development (Zhu et al., 2023). Mechanistically, OTUB1 binds FGF receptor 2 (FGFR2) and decreases its ubiquitination through the non-canonical mechanism (Zhu et al., 2023). Interestingly, OTUB1 inhibits FGFR2 degradation through lysosome, consistent with FGFR2 being a membrane protein. FGFR2 mediates the activation of ERK and PI3K pathways in osteoblasts. Introducing exogenous FGFR2 with adeno-associated viruses restores osteoblastic bone formation (Zhu et al., 2023).

### 4.2 OTUB1 in cancer

**FIGURE 4:**
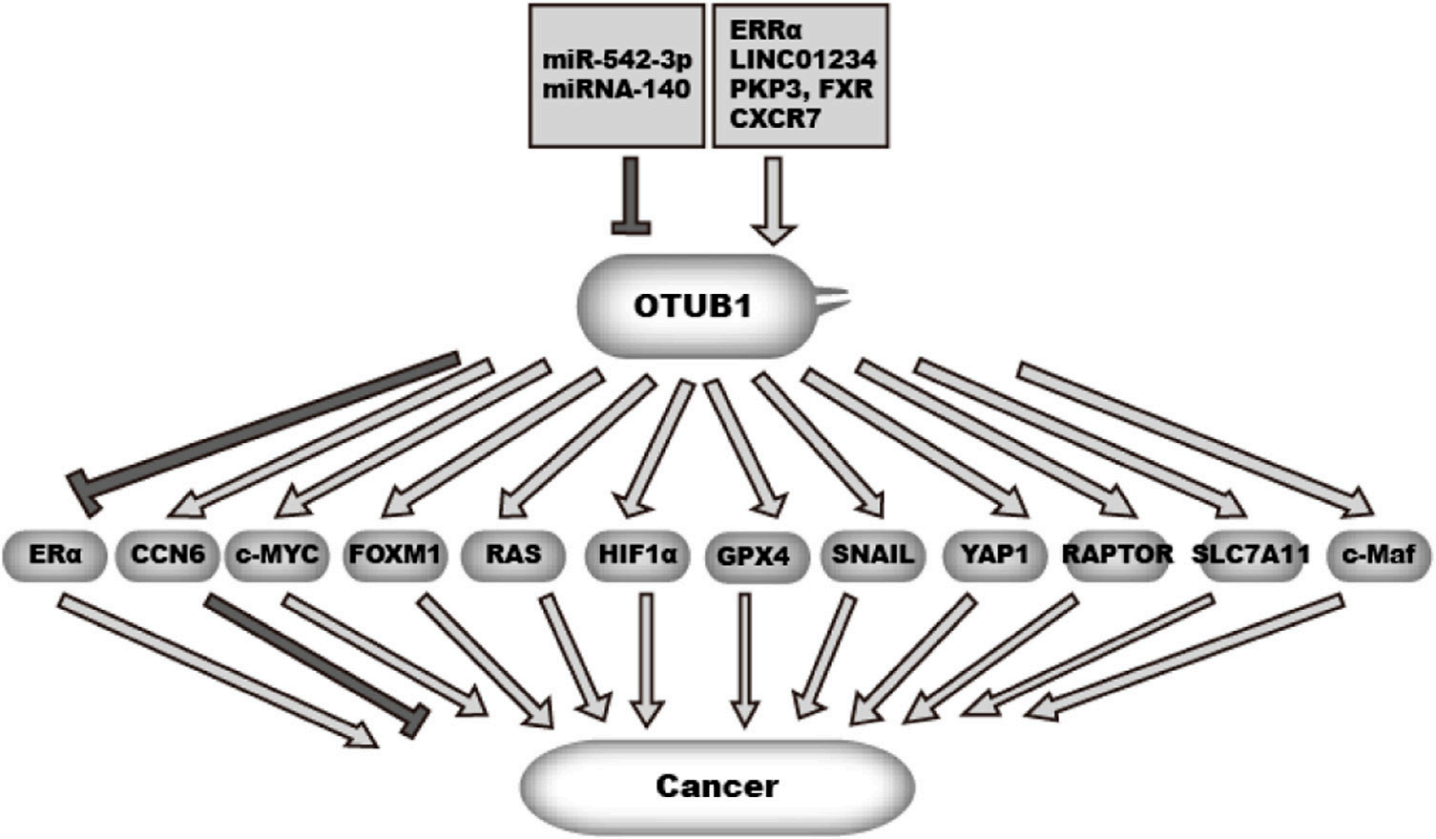
Function and regulation of OTUB1 in cancer.

Cancer originates from uncontrolled cell proliferation. In addition, cancer cells need to acquire the ability to metastasize and escape immune surveillance. To gain these abilities, cancer rewires gene expression, cellular metabolism, and cell cycle control and increases cell motility (Hanahan and Weinberg, 2011). Additionally, cancer cells need to survive or adapt to various stresses and anti-cancer therapy. OTUB1 is overexpressed in many cancer types (Figure 4). Studies have shed insight into the mechanism underlying OTUB1 overexpression (Figure 4). Zhou et al. found estrogen-related receptor alpha (ERRα) binds to the OTUB1 promoter and promotes its transcription (Zhou et al., 2019b). In addition, microRNA miR-542-3p, often downregulated in cancer, can target OTUB1 mRNA (Yuan et al., 2017; Sun et al., 2020). Xie et al. (2019) found LncRNA LINC01234 increases OTUB1 expression by sponging miRNA miR-140 while LINC01234 itself is highly expressed in lung cancer. RNA binding proteins PKP3 (plakophilin 3) and FXR bind and stabilize OTUB1 mRNA (Liu et al., 2021). In breast cancer, chemokine (C-X-C motif) receptor 7 (CXCR7) is overexpressed. Hao et al. found CXCR7 upregulates OTUB1 through ERK1/2 (Hao et al., 2018). In addition, the mTOR pathway upregulates OTUB1 expression in CD4^+^ T cells. mTOR is among the most frequently upregulated pathways in cancer. But it is not clear whether this mechanism also works in cancer cells (Lin et al., 2009). Functionally, OTUB1 affects many cancer-related processes through various effectors (Figure 4).

OTUB1 expression is regulated by multiple factors in cancer, including non-coding RNA and transcription factors.OTUB1 in turn can either promote or suppress malignancy in cancer through deubiquitinating multiple effectors. 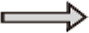
denotes a promoting effect while 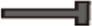
denotes an inhibiting effect.

#### 4.2.1 OTUB1 in breast cancer and gynecological cancer

##### 4.2.1.1 OTUB1 stabilizes c-MYC in breast cancer

Han et al. (2022) set out to dissect the potential role of OTUB1 in breast cancer cells. They identified c-MYC in OTUB1 immunoprecipitate with mass spectrometry. C-MYC is a master transcription factor and a proto-oncogene. The authors further showed OTUB1 interacts with and directly deubiquitinates c-MYC at lysine-K323. Consequently, OTUB1 increases c-MYC protein levels and the expression of c-MYC target genes including hexokinase-2, which increase glycolysis and tumor growth. Consistently, silencing OTUB1 decreases breast cancer growth which can be counteracted by c-Myc overexpression in breast cancer cells MCF7 and MDA-MB-231 (Han et al., 2022). As the activation of c-MYC contributes to the progression of multiple types of cancer, this study implies a general tumor-promoting role of OTUB1.

##### 4.2.1.2 OTUB1 stabilizes FOXM1 in breast and ovarian cancer

FOXM1 is a forkhead transcription factor involved in proliferation, metastasis, and drug resistance in cancer (Myatt and Lam, 2008; Katzenellenbogen et al., 2023). Karunarathna et al. (2016); Wang et al. (2016) independently found FOXM1 interacts with OTUB1. They further showed OTUB1 decreases FOXM1 ubiquitination and promotes FOXM1 protein stability. Wang et al. (2016) also provided evidence that OTUB1 directly removes K48-linked ubiquitination from FOXM1. Katzenellenbogen et al. (2023) showed OTUB1 promotes breast cancer cell proliferation through FOXM1. In addition, FOXM1 is known to promote cellular resistance to epirubicin, a genotoxic drug used to treat breast cancer. In their study, Karunarathna et al. showed OTUB1 also promotes breast cancer resistance to epirubicin. In parallel, Wang et al. showed OTUB1 promotes cancer cell proliferation and migration in ovarian cancer cells. Both studies found a correlation between OTUB1 and FOXM1 protein levels in cancer samples.

##### 4.2.1.3 OTUB1 inhibits estrogen receptor α (ERα) function in endometrial cancer

The DUB activity of OTUB1 was demonstrated for polyubiquitin chains assembled *in vitro* in 2003 (Balakirev et al., 2003), but its substrate *in vivo* was not identified until a few years later. ERα is a major mediator of estrogen function involved in breast cancer and endometrial cancer. ERα inhibitors have been the pillar in breast cancer therapy. ERα, upon bound by estrogen, translocates to the cell nucleus and activates transcription of a wide spectrum of genes, which promotes cancer cell proliferation. Interested in regulatory mechanism of ERα, Stanisic et al. (2009) performed mass spectrometry analysis of ERα immunoprecipitate and identified OTUB1 as a potential interaction partner of ERα. Further study showed OTUB1 directly deubiquitinates ERα and decreases its catalytic activity in Ishikawa endometrial cancer cells. Mechanistically, OTUB1 increases ERα in the chromatin-bound fraction, which implies OTUB1 represses ERα motility required for its activity. This work hints at a tumor-inhibiting role of OTUB1 in ER-positive breast cancer and endometrial cancer. Nevertheless, such possibility was not directly examined in this study (Stanisic et al., 2009).

##### 4.2.1.4 OTUB1 stabilizes CCN6 in breast cancer

CCN6 is a breast cancer tumor suppressor gene that encodes a secreted protein. The CCN6 gene is lost in some advanced breast cancer (van Golen et al., 1999) while CCN6 knockout mice develop breast cancer (Martin et al., 2017). CCN6 inhibits both the proliferation and metastasis of breast cancer cells (Kleer, 2016). Mechanistic studies found CCN6 inhibits the expression of SNAIL and ZEB1 (Kleer, 2016). In addition, CCN6 inhibits the effects of BMP4 (bone morphogenetic protein 4), TGFβ (transforming growth factor β), and IGF-1 (insulin-like growth factor 1) (Kleer, 2016). Zhao et al. (2023) investigated the regulation of CCN6 protein levels. Through overexpressing a panel of deubiquitinases, they identified that OTUB1 overexpression upregulates CCN6 protein levels. Further study showed OTUB1 interacts with CCN6 and decreases CCN6 ubiquitination independent of its enzymatic activity (Zhao et al., 2023). The authors showed OTUB1 knockout increases the proliferation and migration of 4T1 mouse breast cancer cells, which can be counteracted by CCN6 expression. This observation is recapitulated in MDA-MB-231 human breast cancer cells with siRNA transfection. Collectively, this study identified a OTUB1–CCN6 signal axis that suppresses cancer cell proliferation and migration in advanced breast cancer.

#### 4.2.2 OTUB1 in lung cancer

##### 4.2.2.1 OTUB1 activates RAS in lung cancer

RAS, a family of proto-oncogenes encoding small G-proteins, include K-RAS, N-RAS, and H-RAS. Constitutive activation of RAS due to point mutation is seen in different cancer types including lung cancer and pancreatic adenocarcinoma (Moore et al., 2020). RAS activates the downstream MAPK pathway at the plasma membrane, which is inhibited by RAS monoubiquitination (Xu et al., 2010). To identify potential DUBs regulating RAS, Baietti et al. performed a protein–protein interaction screening (Baietti et al., 2016). They identified OTUB1 interacts with RAS. OTUB1 inhibits RAS ubiquitination which is independent of DUB activity as the C91S mutant has similar effect. As a result, OTUB1 increases RAS localization to the plasma membrane and the activation of the downstream MAPK pathway (Baietti et al., 2016). OTUB1 gene amplification in lung cancer is exclusive with RAS activating mutation, indicating OTUB1 is a driving force for the activation of wildtype RAS (Baietti et al., 2016). Consistently, RAS-wildtype lung cancer cells tend to be more dependent on OTUB1. OTUB1 levels have a bigger impact on the survival of RAS-wildtype patients than Ras-mutant patients in the TCGA (The Cancer Genome Atlas) cohort (Baietti et al., 2016).

##### 4.2.2.2 OTUB1 stabilizes HIF1α in lung cancer

HIF1α is a master regulator of cellular response to hypoxia. Aberrant activation of HIF1α contributes to the proliferation, angiogenesis, and metastasis of cancer cells (Keith et al., 2011; Harris et al., 2022). HIF1α protein stability is reined by PHD-mediated hydroxylation followed by VHL-mediated ubiquitination under normoxia (Keith et al., 2011). Liu et al. (2022) set out to identify additional regulators of HIF1α. They found OTUB1 interacts with HIF1α and decreases the K48-linked ubiquitination of HIF1α in H1299 human lung cancer cells. OTUB1 expression is higher in lung cancer than in normal samples in the TCGA cohort. In addition, positive correlation exists between OTUB1 and GLUT1, a typical HIF1α target gene. This effect of OTUB1 on HIF1α is through the non-canonical mechanism of OTUB1 as it is sensitive to D88A mutation but not C91A mutation. Overexpressing OTUB1 augments hypoxia gene induction while knocking down OTUB1 has the opposite effect (Liu et al., 2022). As a result, OTUB1 facilitates cancer adaption to hypoxia. OTUB1 knockout lung cancer cells proliferate much slower under hypoxia than control cells with decreased glycolysis and glucose uptake.

##### 4.2.2.3 OTUB1 stabilizes mitochondrial proteins in lung cancer

A study by Sheryazdanova et al. (2023) identified another mechanism by which OTUB1 promotes lung cancer cell proliferation. They failed to detect the effect of OTUB1 on HIF1α protein stability. Instead, they found hypoxia changes the interactome of OTUB1 protein in A549 lung cancer cells, reminiscent of a previous report (Scholz et al., 2016). Sheryazdanova et al. found mitochondrial proteins are significantly enriched in the OTUB1 interactome. Importantly, OTUB1 knockdown significantly decreases mitochondrial protein levels and increases their ubiquitination. Functionally, OTUB1 promotes the oxidative phosphorylation of lung cancer cells. In contrast, OTUB1 knockdown decreases oxidative phosphorylation of lung cancer cells and inhibits cell proliferation.

#### 4.2.3 OTUB1 in esophageal cancer

##### 4.2.3.1 OTUB1 stabilizes SNAIL in esophageal cancer

SNAIL is a key transcription factor driving cell migration and invasion in cancer cells. To identify DUBs regulating SNAIL, Zhou et al. (2018) expressed a panel of DUBs in HEK-293T cells to examine their effect on SNAIL protein levels. They found OTUB1 increases SNAIL protein levels. Further study showed that OTUB1 interacts with SNAIL, directly deubiquitinates SNAIL, and promotes SNAIL protein stability (Zhou et al., 2018). OTUB1 is overexpressed in esophageal squamous cell carcinoma and correlates with the level of SNAIL (Zhou et al., 2018). Functionally, OTUB1 promotes cell invasion *in vitro* through SNAIL. With a tail-vein injection model, the authors showed OTUB1 promotes lung metastasis of esophageal squamous cell carcinoma cells *in vivo* (Zhou et al., 2018).

#### 4.2.4 OTUB1 in gastric cancer

Weng et al. (2016) performed immunohistochemistry analysis on gastric cancer patient samples and found OTUB1 was overexpressed in gastric cancer samples compared to normal samples and intraepethelial neoplasia. *In vitro* study showed OTUB1 promotes gastric cancer cell migration and invasion. But the mechanism remained elusive at that time. Two later studies provided mechanistic insight into the function of OTUB1 in gastric cancer.

##### 4.2.4.1 OTUB1 stabilizes YAP1 in gastric cancer

YAP1 is a transcription factor which contributes to tumor progression in multiple cancer types (Moroishi et al., 2015; Zanconato et al., 2019). Interested in YAP1 regulation in gastric cancer, Yan et al. (2022) performed a siRNA (small interfering RNA) screen for deubiquitinase that could inhibit YAP1 ubiquitination. They found OTUB1 decreases YAP1 ubiquitination and promotes YAP1 protein stability. The effect is through the non-canonical mechanism of OTUB1 as it is abolished by the D88A mutation but not the C91S mutation. In addition, the authors showed OTUB1 interacts with YAP1. As a result, OTUB1 promotes gastric cancer cell proliferation, migration, and invasion as well as stemness through YAP1. To corroborate the clinical relevance, the authors showed OTUB1 is overexpressed in gastric cancer and correlates with YAP activity.

##### 4.2.4.2 OTUB1 stabilizes GPX4 in gastric cancer

A study by Li et al. identified another mechanism by which OTUB1 promotes gastric cancer progression. The authors showed OTUB1 inhibits ferroptosis in gastric cancer and promotes cell proliferation as well as migration and invasion. Ferroptosis is a type of cell death induced by the peroxidation of membrane lipids (Lei et al., 2022). Cells employs multiple mechanisms to combat lipid peroxidation, among which GPX4 (glutathione peroxidase 4) plays a key role. GPX4 reduces oxidized lipid back to the reduced state with glutathione as the reducing agent. Mechanistically, Li et al. (2023a) showed OTUB1 is recruited to GPX4 by a protein called CST1, inhibiting GPX4 ubiquitination and degradation.

#### 4.2.5 Genitourinary cancer

Kidney cancer, bladder cancer, and prostate cancer are the most common types of genitourinary cancer. Studies have revealed relationships between these cancer types and OTUB1.

##### 4.2.5.1 OTUB1 stabilizes FOXM1 in renal cell carcinoma (RCC)

RCC is the most common type of kidney cancer. Zhou et al. (2020) found OTUB1 is overexpressed in RCC tissues and cell lines, which is associated with the poor prognosis of RCC patients. Knocking down OTUB1 inhibits cell viability and proliferation as well as migration and invasion. FOXM1, a previously identified substrate of OTUB1 (Wang et al., 2016), mediates this effect of OTUB1 in RCC. Overexpressing FOXM1 rescues the defects in OTUB1 knockdown RCC cells. The authors show FOXM1 upregulates the expression of ECT2 (epithelial cell transforming 2), which mediates the effect of OTUB1 and FOXM1 (Zhou et al., 2020).

##### 4.2.5.2 OTUB1 regulates mTOR in RCC

A study by Seo et al. revealed a second mechanism by which OTUB1 promotes kidney cancer. MTORC1 complex is a key protein kinase that promotes cell growth (Goul et al., 2023). Overactivation of mTORC1 is common in cancer. **RAPTOR** is a core subunit of the mTORC1 complex necessary for its kinase activity (Linde-Garelli and Rogala, 2023; Panwar et al., 2023). Interested in the regulation of RAPTOR, Seo et al. (2023) performed shRNA (short-hairpin RNA) screening for DUBs that could regulate RAPTOR expression. They found OTUB1 upregulates RAPTOR protein levels. OTUB1 interacts with RAPTOR and increases its protein stability (Seo et al., 2023). OTUB1 decreases RAPTOR ubiquitination which is independent of DUB activity as C91S mutants still have the effect. In addition, D88A mutants do not affect this effect either. Instead, the effect of OTUB1 is dependent on intact Tyrosine-26 (Y26) and the authors showed Y26 phosphorylation is required for OTUB1-RAPTOR interaction. Consistently, the Y26A mutation decreases interaction between OTUB1 and RAPTOR. As a result, OTUB1 is required for nutrient-induced mTORC1 activation. In addition, OTUB1 also increases the kidney cancer cell tolerance of chemotherapeutic drugs oxaliplatin and doxorubicin through RAPTOR. RAPTOR is a key mediator of these functions of OTUB1 as RAPTOR overexpression rescues the defect in OTUB1-KD cells. The authors found Y26 is phosphorylated by non-receptor tyrosine kinases, Src, and SRMS, which is detected at high levels in renal cell carcinoma. In kidney cancer patient samples, OTUB1-Y26 phosphorylation is significantly increased and correlates with RAPTOR protein levels. This study collectively delineated a Src-OTUB1–mTORC1 axis in kidney cancer. Interestingly, another study revealed another layer of regulation of mTOR by OTUB1. Zhao et al. found OTUB1 interacts with DEPTOR, which is a negative regulator of mTORC1 activity. They found OTUB1 increases DEPTOR protein stability. This activity is independent of OTUB1’s DUB activity but requires intact D88. As a result, OTUB1 decreases mTORC1 activity. Nevertheless, the effect of OTUB1 on DEPTOR was shown in HEK293T and HeLa cells and the biological consequences in human cancer require further investigation (Zhao et al., 2018).

##### 4.2.5.3 OTUB1 stabilizes SLC7A11 in bladder cancer

Cystine can be utilized to generate cysteine and glutathione, which are important for cellular redox balance. Dysregulation of redox balance may lead to ferroptosis, a type of cell death caused by lipid peroxidation. SLC7A11 is a key component of the cystine-glutamate antiporter, which exchanges intracellular L-glutamate for extracellular L-cystine. SLC7A11 thus plays a critical role in cellular sensitivity to ferroptosis. Interested in the regulation of SLC7A11, Liu et al. (2019) performed mass spectrometry to identify proteins interacting with SLC7A11. They identified OTUB1 as an interaction partner of SLC7A11. Further study showed OTUB1 reduces SLC7A11 ubiquitination, which is through the non-canonical mechanism of OTUB1 as C91S mutants, but not D88A, still have the effect. The authors found OTUB1 is overexpressed in bladder cancer. Loss of OTUB1 inhibits bladder tumor growth and leads to ferroptosis. Interestingly, two studies have revealed negative feedback mechanisms of OTUB1 regulation by redox balance (Chen et al., 2021; Aboushousha et al., 2023). Chen et al. found redox increases OTUB1 persulfidation, which increases interaction with SLC7A11 and increases cystine import to combat redox stress and ferroptosis (Chen et al., 2021). On the other hand, Aboushousha et al. found redox increases OTUB1 S-glutathionylation, which increases interaction with E2 and increases SLC7A11 stabiity (Aboushousha et al., 2023).

##### 4.2.5.4 OTUB1 promotes malignancy of prostate cancer

OTUB1 also functions in prostate cancer cells. Iglesias-Gato et al. (2015) screened for the effects of OTU-family DUBs on prostate cancer cell proliferation, migration, and invasion with the siRNA library. They found that OTUB1 promotes the invasion and tumorigenesis of prostate cancer *in vitro* and *in vivo* by promoting Rho-A activation. In addition, Liao et al. (2020) also found OTUB1 promotes the progression and proliferation of prostate cancer cells. They suggest cyclin E3 be stabilized and deubiquitinated by OTUB1.

#### 4.2.6 Multiple myeloma

##### 4.2.6.1 OTUB1 stabilizes c-MAF in multiple myeloma

C-Maf is a transcription factor encoded by a pro-oncogene for multiple myeloma. C-Maf is overexpressed in multiple myeloma and correlates with poor prognosis in multiple myeloma patients (Zhan et al., 2006). It upregulates genes including cyclin D2 and integrin β7 to promote cell proliferation and survival (Hurt et al., 2004). c-Maf also leads to multiple myeloma resistance to proteasome inhibitors. Interested in the regulation of c-Maf, Xu et al. (2021) performed affinity purification of HA-tagged c-Maf and performed mass spectrometry to identify c-Maf interactome. They identified that OTUB1 interacts with c-Maf. They showed OTUB1 decreases c-maf ubiquitination and increases its protein stability. This activity is independent of OTUB1’s DUB activity. Moreover, the D88A mutant of OTUB1 still stabilizes c-Maf protein. But this effect of OTUB1 can be inhibited by OTUB1 N-terminal deletion. As a result, inhibiting OTUB1 decreases c-Maf protein levels and causes cell apoptosis in multiple myeloma cells (Xu et al., 2021).

##### 4.2.6.2 Summary of OTUB1 functions in cancer

As discussed above, numerous studies have shown OTUB1 plays a prominent role in cancer. While most studies support a pro-tumor role of OTUB1, the conclusion is not unanimous. There are a few explanations underlying this contradiction. Firstly, it could be due to different biologies in different cancer types. Secondly, many of these studies are based on cancer cell lines. The conclusions could be confounded by potential differences in the cell lines used or long-time sub-culture of the same cell lines. Thirdly, there are differences in methodology. For example, a single colony was used in one study (Zhao et al., 2023) while a cell population was used in another study (Han et al., 2022). For genetic intervention, CRISPR/Cas9-mediated knockout, synthetic siRNA, or lentivirus-expressed shRNA were used. All these differences could potentially affect the conclusions. These discrepancies need to be clarified with further studies on more clinically relevant models including genetic-modified mouse models and patient-derived xenografts. These discrepancies may also indicate patients need to be stratified for future therapeutics targeting OTUB1.

### 4.3 OTUB1 in DNA damage response

**FIGURE 5:**
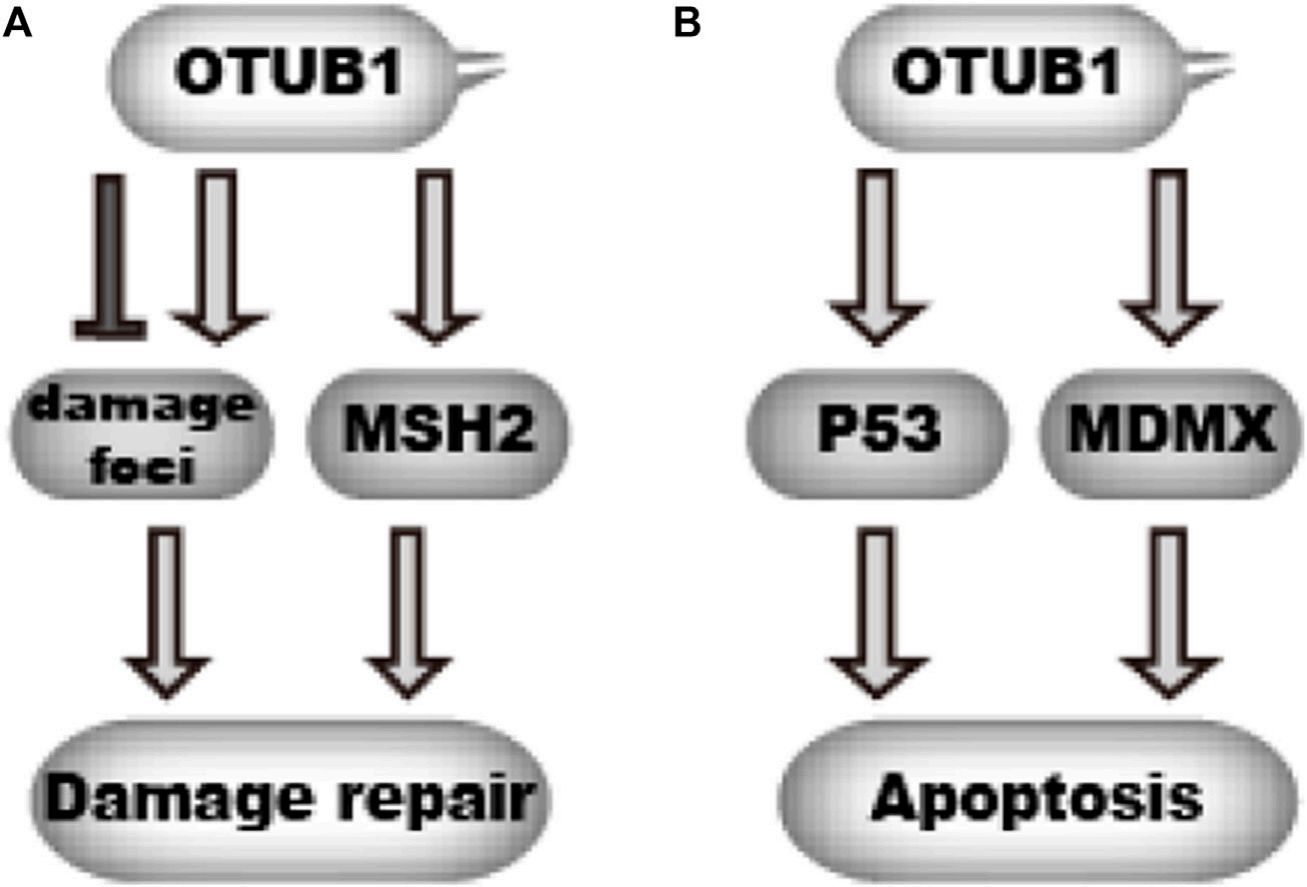
OTUB1 regulates DNA damage response **(A)**.OTUB1 can either promote or delay DNA damage repair through targeting DNA damage foci and MSH2. 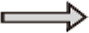
denotes a promoting effect while 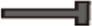
denotes an inhibiting effect. **(B)** By inhibiting ubiquitination of P53 and MDMX, OTUB1 can promote cellular apoptosis upon DNA damage. 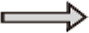
denotes a promoting effect while 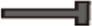
denotes an inhibiting effect.

Genomic DNA is constantly challenged and damaged by endogenous and exogenous threats. Cells utilize multiple mechanisms to repair DNA damage, collectively called DNA damage repair (DDR). DDR is critical for genome stability. Unrepaired damage increases the likelihood of cancer. For all the pathways of DNA damage repair, cells treat the damage as a signal. Cells recognize the damage and summon a cascade of events. During these cascades, protein machinery are recruited to the damage foci and relieved in an orderly manner to repair the damage. Prompt and reversible protein post-translational modifications play a key role in this process. Protein ubiquitination is one type of the modification that is critical for DNA damage repair, which is best shown in the repair of double-strand breaks (Jackson and Durocher, 2013). Previous studies reveal a prominent role of OTUB1 in the reversible ubiquitination during DNA damage repair (Figure 5A). Alternatively, excessive DNA damage may cause cell apoptosis, during which process P53 is a key controller (Vucic et al., 2011). OTUB1 regulates P53-dependent apoptosis after DNA damage as well (Figure 5B).

#### 4.3.1 OTUB1 functions in resolving of DNA damage foci

After DNA damage, a wave of ubiquitination events occur on DNA damage foci, which play a critical role in DNA damage repair (Jackson and Durocher, 2013). RNF8 and RNF168 are key E3 ligases involved in this process. But the ubiquitination needs to be removed as the damage gets resolved. To uncover potential DUBs involved, Nakada et al. screened for DUBs with siRNA. They found OTUB1 counteracts the ubiquitination at damage foci as knocking down OTUB1 leads to persistent ubiquitination at damage foci and delayed DNA repair (Figure 5A). However, overexpressing OTUB1 also hinders DNA damage repair as it prevents proper accumulation of ubiquitination at damage foci. Intriguingly, mutation of C91, the catalytic cysteine, does not abolish the effect of OTUB1. Instead, D88A/H265A mutation or N-terminus deletion abolishes the effect of OTUB1, which for the first time indicates OTUB1 can function through a non-canonical mechanism independent of DUB activity. To reveal the underlying mechanism, the authors performed mass spectrometry analysis for OTUB1 immunoprecipitate and found Ubc13 (UBE2N), UBE2D and UBE2E E2s interact with OTUB1. Further biochemical study found OTUB1 inhibits ubiquitin transfer from E2s to acceptor ubiquitin, thus blocking the formation of poly-ubiquitin chains. Of note, UBE2N preferentially generates the K63-linked ubiquitin chain, which shows the specificity for K48-linked chains in the canonical mechanism does not apply to the non-canonical mechanism (Nakada et al., 2010). Later, several structural studies independently revealed how OTUB1 inhibits ubiquitin transfer from E2. The N-terminal alpha-helix of OTUB1 engages the E2∼ubiquitin complex and locks it in an inactive conformation. Unexpectedly, free ubiquitin facilitates the binding between OTUB1 and E2∼ubiquitin conjugate (Juang et al., 2012; Sun et al., 2012; Wiener et al., 2012).

#### 4.3.2 OTUB1 stabilizes MSH2

Wu et al. revealed another mechanism by which OTUB1 contributes to DNA damage repair. Mismatch repair corrects DNA replication errors which occurs constantly in proliferating cells. MSH2 is involved in the recognition of DNA replication error to initiate DNA mismatch repair. A too high or too low level of MSH2 can increase mutation frequency (Shcherbakova and Kunkel, 1999), which means MSH2 protein levels must be under tight regulation. Interested in the potential mechanism of MSH2 protein regulation, Wu et al. found that OTUB1 interacts with MSH2 and increases MSH2 protein stability (Wu et al., 2021). Mechanistically, OTUB1 inhibits MSH2 ubiquitination by blocking ubiquitin transfer from E2 (Figure 5A). As a result, the loss of OTUB1 decreases MSH2 protein levels and leads to greater mutation frequency.

#### 4.3.3 OTUB1 stabilizes P53 and MDMX

P53 is a master regulator of DNA damage response and cell apoptosis. Upon DNA damage, P53 protein becomes stabilized and activates the transcription of target genes, which promotes cell cycle arrest or apoptosis. P53 protein levels are significantly regulated by the ubiquitin-proteasome pathway. Sun et al. (2012) examined the effect of OTU-family DUBs on P53 expression. They found OTUB1 overexpression increases P53 protein levels. Further study revealed that OTUB1 interacts with P53 and decreases its ubiquitination (Figure 5B). This effect is independent of OTUB1’s DUB activity. Instead, OTUB1 binds and inhibits UbcH5 (UBE2D family), the cognate E2 for MDM2 which is a key E3 ligase for P53. Mutation of D88 to alanine disrupts this effect of OTUB1. Consistent with its effect on P53, OTUB1 promotes cell apoptosis after DNA damage in P53-Wildtype cells. The authors later showed OTUB1 also increases MDMX protein levels through a similar mechanism, which facilitates P53 activation (Chen et al., 2017) (Figure 5B). Altogether, OTUB1 increases P53 protein level and promotes DNA-damage-induced apoptosis in P53-Wildtype cells. Nevertheless, it is noteworthy the effect of OTUB1 on apoptosis might be confounded by the effect of OTUB1 on DNA damage repair.

DNA damage repair is intimately connected to cancer. Failure in DNA damage repair increases cancer incidence. In contrast, excessive DNA damage causes cell death in not only normal cells but also cancer cells. In fact, many chemotherapy regimens and radiotherapy kill cancer cells through DNA damage. From the perspective of DNA damage repair, the effect of OTUB1 on DNA damage might indicate a bipartite role of OTUB1 in cancer. Firstly, OTUB1 contributes to genomic stability and prevents cancer occurrence. Secondly, OTUB1 might affect the cancer cell sensitivity to DNA damaging agents.

### 4.4 OTUB1 in immune response

**FIGURE 6:**
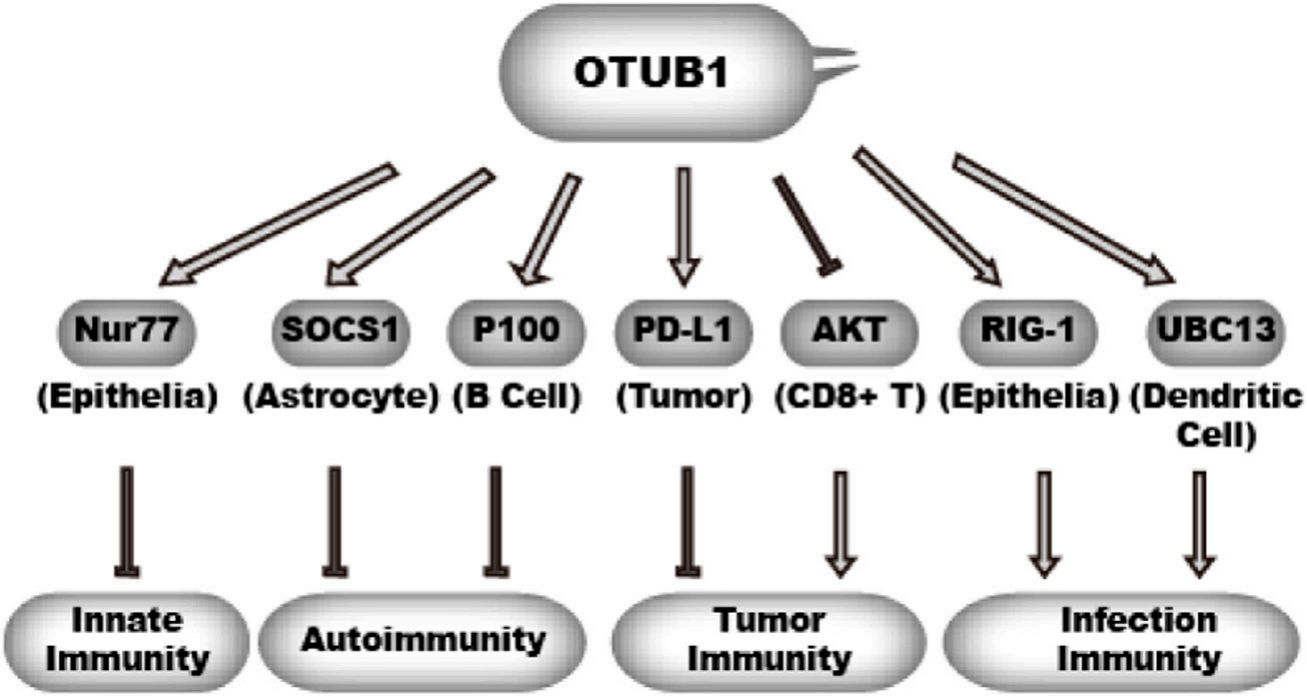
OTUB1 in immunity.

Immune response serves as a guardian against infectious agents and mutated host cells. Immune response has to be tightly regulated by both positive and negative mechanisms. Excessive immune activity gives rise to inflammatory diseases and autoimmune diseases. The activity and stability of immune modulatory proteins are subject to regulation by ubiquitination. Consistently, studies in the past have uncovered a multifaceted role of OTUB1 in immune response (Figure 6). There is evidence that OTUB1 represses anti-tumor immunity, innate immunity, and autoimmunity. However, evidence also indicates OTUB1 may promote anti-infection immunity.

OTUB1 inhibits ubiquitination of different effectors in different cell types, which in turn either promotes immunity or inhibits immunity. 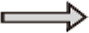
denotes a promoting effect while 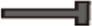
denotes an inhibiting effect.

#### 4.4.1 OTUB1 suppresses tumor immunity

Cytotoxic CD8^+^ T cells are at the frontline of fighting cancer. CD8^+^ T cells directly recognize and kill cancer cells, which are considered to be the most important mechanisms in tumor immunity. However, cancer cells manage to escape the immune surveillance of CD8^+^ T cells, which involves multiple mechanisms (Spranger and Gajewski, 2018). PD-L1 is a prominent immune-suppressive molecule on tumor cells as part of the immune-checkpoint (Kraehenbuehl et al., 2022). Through binding PD-1 on cytotoxic T-cells, PD-L1 inhibits the anti-tumor activity of cytotoxic T-cells. PD-L1 expression levels are regulated by ubiquitin-proteasome-mediated degradation. Zhu et al. (2021a) investigated whether OTU-family DUBs could regulate PD-L1 expression. They knocked down different OTU-family DUBs and found OTUB1 knockdown significantly decreases PD-L1 protein levels in breast cancer cells. They extended this finding to other cancer cells. OTUB1 interacts with PD-L1 and directly removes K48-linked ubiquitin chains from PD-L1. As a result, silencing OTUB1 increases tumor sensitivity to T-cell mediated cytotoxicity. In addition, OTUB1 inhibits tumor immune surveillance by another mechanism. To study the function of OTUB1 in T-cells, Zhou et al. (2019a) deleted OTUB1 in T-cells and found OTUB1 loss causes aberrant activation of CD8^+^ T cells. Consistently, loss of OTUB1 increases the anticancer immunity of CD8^+^ T cells. IL-15 can promote the activation of CD8^+^ T cells. However, IL-15 also triggers OTUB1 to partially redistribute to the plasma membrane where OTUB1 represses the ubiquitination-dependent activation of AKT. AKT is critical for T-cell activity. As a result, OTUB1 represses T-cell response to IL-15. This activity of OTUB1 is independent of its DUB activity as C91S mutants are still effective (Zhou et al., 2019a).

#### 4.4.2 OTUB1 in innate immunity, autoimmunity, and infection immunity

As in the case for tumor immunity, there is evidence showing OTUB1 also represses innate immunity. Nur77 is an orphan nuclear receptor with a transcription activation domain in its N-terminus. Nur77 is expressed in myeloid cells, lymphoid cells, and epithelial cells (Lith and de Vries, 2021). Nur77 inhibits innate immunity and inflammation at least partially through inhibiting NF-κB activity (Lith and de Vries, 2021). Knockdown of Nur77 in human lung epithelial cells results in a marked increase in IκBα phosphorylation, corresponding with elevated NF-κB activity (Kurakula et al., 2015). In inflammatory bowel disease, Nur77 also inhibits NF-κB activity (Wu et al., 2016). Pei et al. found that Nur77 interacts with OTUB1 and is stabilized by OTUB1 (Pei et al., 2019), which is through the non-canonical mechanism sensitive to D88A rather than the C91S mutation. Functionally, the authors showed OTUB1 represses cell response to TNF-α in HeLa cells. Another study also identified a mechanism by which OTUB1 inhibits autoimmunity in astrocytes (Wang et al., 2019b). Wang et al. found astrocyte-specific OTUB1 ablation causes more severe autoimmunity in a multiple sclerosis mouse model. They showed OTUB1 decreases SOCS1 ubiquitination and increases its protein stability. SOCS1 is a key inhibitor of JAK-STAT1 signaling. A decrease in SOCS1 level leads to increased JAK1-STAT1 activity and cytokine production by astrocytes. This activity of OTUB1 is independent of DUB activity but requires its N-terminus.

Li et al. (2019) identified another mechanism by which OTUB1 represses the NF-κB pathway. P100 is an inhibitor of the non-canonical NF-κB pathway, similar to the role of IκB in canonical NF-κB. To identify potential regulators of P100, Li et al. (2019) performed proximity-dependent biotin identification (BioID) assay and identified OTUB1 as a potential P100 interaction partner. They further found OTUB1 decreases P100 ubiquitination and increases P100 protein level. This activity is independent of OTUB1’s DUB activity but requires intact D88 residue. The deletion of OTUB1 in B cell results in B-cell hyperplasia, increased antibody production, increased IL-6 production, and lupus-like autoimmunity. Unlike the cases in astrocytes and epithelial cells, OTUB1 is reported to activate NF-kB in dendritic cells upon infection or LPS (lipopolysaccharide) stimulation. Mulas et al. (2021) found OTUB1 expression is upregulated in dendritic cells by infection or LPS treatment. They showed OTUB1 decreases UBC13 ubiquitination and increases its protein level. UBC13 is an E2 critical for K63-linked ubiquitin chain formation on substrates including IRAK1 and TRAF6 which are critical in innate immune defense. This effect of OTUB1 is dependent on its catalytic activity and promotes immune response in dendritic cells (Mulas et al., 2021). Another work showed after RNA virus infection, OTUB1 in epithelial cells could co-localize with RIG-1 at mitochondria membrane (Jahan et al., 2020). Upon binding to double-stranded RNA (dsRNA), RIG-I transforms from an auto-repressed conformation to an open one, which allows its ubiquitination and tetramerization. The RIG-I tetramer translocates to mitochondrial membrane, where it activates downstream effectors including IRF3, IRF7, and NF-kB to produce type-I Interferon (IFN-I) (Zheng et al., 2023). Jahan et al. (2020) found OTUB1 activates RIG-I at mitochondria and achieves this function through both DUB activity and E2-blocking activity. As a result, OTUB1 deletion causes defects in NF-κB and IRFs (interferon regulatory factor) responses after RNA virus infection. Interestingly, type-I interferons, for example, INF-β, induce expression of OTUB1, which implies a positive feedback between OTUB1 and RIG-I-mediated innate immunity during RNA virus infection. However, influenza A virus can trigger OTU1 degradation to sabotage this anti-infection mechanism.

Collectively, these result suggest OTUB1 might suppress the immune response to host cells including tumor cells or normal cells. In contrast, OTUB1 might be summoned to activate the immune response to infectious pathogens.

### 4.5 Other functions of OTUB1

OTUB1 also functions in biological processes other than those elaborated above. Herhaus et al. (2013) identified OTUB1 increases the protein stability of SMAD3 and promotes cell migration in skin cells induced by TGF-β. But whether this mechanism can be extended to human diseases is unknown. In addition, OTUB1 can increase Tau accumulation in Alzheimer’s diseases, which aggravates Alzheimer’s diseases (Wang et al., 2017). In Parkinson’s disease, redox stress causes the S-Nitrosylation of OTUB1, which inhibits its function (Kumari et al., 2022). It is reasonable to believe more biological functions of OTUB1 will be uncovered in the future.

## 5 OTUB1 as a therapeutic target

Despite many tremendous advancements, cancer therapy still faces insufficient cancer targets and drug resistance. Previous studies have shown OTUB1 contributes to cancer cell survival, proliferation, migration, and invasion. In addition, OTUB1 affects tumor sensitivity to current therapies. These findings indicate OTUB1 could be a promising target in cancer therapy.

### 5.1 Rationale for OTUB1 inhibitor as monotherapy

Multiple studies have shown OTUB1 promotes cancer cell proliferation. Therefore, inhibiting OTUB1 may retard tumor growth or cause cell death. Two lines of efforts have testified for such a possibility. As OTUB1 is critical for multiple myeloma cell survival by stabilizing c-MAF protein, Xu et al. (2021) carried out a small molecule screen in multiple myeloma cells that was based on the c-Maf-dependent luciferase reporter. They found the natural product lanatoside C inhibits OTUB1’s effect on c-MAF. Accordingly, this compound induces apoptosis in multiple myeloma cells at nanomolar dosages (Xu et al., 2021). Through similar methods, the herbal acevaltrate and anti-bacterial/anti-viral nanchangmycin have been identified to inhibit OTUB1/c-MAF axis and induce myeloma cell apoptosis (Xu et al., 2020; Sun et al., 2021). More recently, Tan et al. (2022) discovered a potent OTUB1/USP8 dual inhibitor by screening with an *in vitro* DUB assay on a fluorescent substrate. The compound inhibits non-small-cell lung cancer proliferation at sub-micromolar dosages. Besides chemical compounds, the therapeutic use of small RNA targeting OTUB1 could be beneficial as well. Ectopic expression of miR-542-3p which inhibits OTUB1 expression, attenuates proliferation, migration, and invasion while promoting apoptosis in colorectal cancer cells (Yuan et al., 2017). Collectively, inhibiting OTUB1 alone may have therapeutic effects in sensitive cancers.

### 5.2 Rationale for using OTUB1 inhibitor in combination therapy

Genetic studies have shown or hinted that OTUB1 inhibitors may have various applications in combination therapy. Estrogen receptor inhibitors are the cornerstone of breast cancer therapy. Tamoxifen has been a first-line drug for decades. Hao et al. (2018) showed OTUB1 increases tamoxifen resistance in breast cancer by stabilizing estrogen receptor-α. Inhibiting OTUB1 promotes sensitivity to tamoxifen. In addition, the early finding that OTUB1 is involved in DNA damage repair indicates OTUB1 might affect cancer cell sensitivity to genotoxic drugs. Consistently, Karunarathna et al. showed suppressing OTUB1 increases the sensitivity of breast cancer to epirubicin (Wang et al., 2016). Alternatively, OTUB1 increases the kidney cancer cell tolerance of chemotherapeutic drugs oxaliplatin and doxorubicin through RAPTOR (Seo et al., 2023). Immune checkpoint inhibitors are used in clinics but many patients respond poorly. Several independent studies indicate OTUB1 inhibitors may increase the efficacy of immune checkpoint inhibitors (Zhu et al., 2021a). Firstly, inhibiting OTUB1 decreases the expression of PD-L1 (Kraehenbuehl et al., 2022). Secondly, OTUB1 represses the activity of CD8^+^ T cells (Zhou et al., 2019a). Thirdly, OTUB1 represses ferroptosis in bladder cancer and gastric cancer (Liu et al., 2019; Li et al., 2023a). Ferroptosis has recently been identified as an important mechanism of cytotoxic T cell-mediated killing (Wang et al., 2019a; Friedmann Angeli et al., 2019). Collectively, inhibiting OTUB1 may increase cancer sensitivity to conventional therapies.

Targeting OTUB1 may not be limited to developing OTUB1 inhibitors. In P53-wildtype cells, OTUB1 may increase cell sensitivity to DNA damage through P53 and MDMX (Sun et al., 2012; Chen et al., 2017). Deubiquitinase-targeting chimera is a recently-developed strategy harnessing DUBs to stabilize a target protein, which has been realized for OTUB1 (Henning et al., 2022). This method may be applied to increase the binding between OTUB1 and wildtype P53 to enforce cancer cell apoptosis.

### 5.3 Perspective for OTUB1-based cancer drug discovery

**FIGURE 7:**
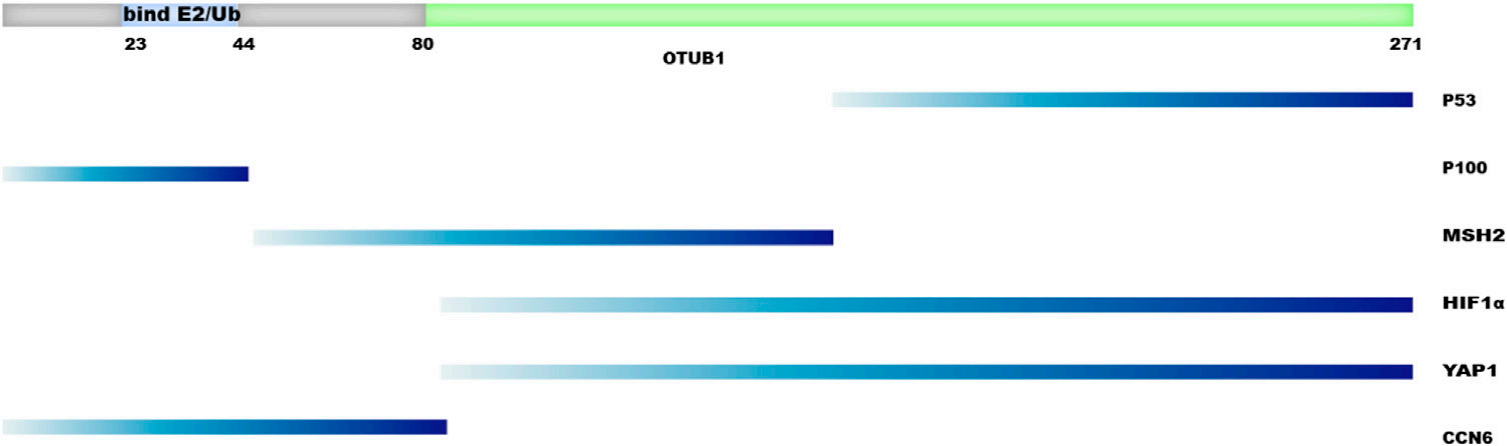
Regions of OTUB1 interacting with other proteins. Regions of OTUB1 are depicted on the left as horizontal bars while the names of proteins interacting with these regions are shown on the right.

When developing OTUB1 inhibitors, it has to be taken into consideration that OTUB1 functions through at least two major mechanisms, which presents both opportunities and challenges. The DUB-activity-based screening adopted by Tan et al. can identify inhibitors of OTUB1’s DUB activity. On the contrary, the strategy adopted by Xu et al. will most likely identify inhibitors disrupting the interaction between OTUB1 and its effectors. Domain mapping was performed for some of the protein–protein interaction involving OTUB1 (Figure 7). The results suggest different proteins engage different regions of OTUB1, even though OTUB1 is a relatively small protein (Figure 7). It is anticipated that inhibitors can be developed that specifically disrupt the interaction between OTUB1 and another protein. Therefore, it may be possible to develop an inhibitor that enhances anti-tumor immunity and meanwhile avoids a boost in auto-immunity. However, the dual mechanisms used by OTUB1 also mean it is hard to inhibit both mechanisms with one compound. For scenarios where inhibiting both mechanisms is desired, proteolysis targeting chimeric (PROTAC) technology may be one option.

## 6 Concluding remarks

OTUB1 has emerged as a versatile deubiquitinase that plays critical roles in physiological and pathophysiological processes including development, cancer progression, immune response, and DNA damage response. OTUB1 changes the stability and/or activity of multiple proteins through canonical deubiquitination or non-canonical ubiquitination blocking. For both mechanisms, the effect of OTUB1 depends on the interaction between OTUB1 and these proteins. OTUB1’s expression, localization, activity, and interaction with others are exquisitely regulated by multiple mechanisms. Due to the prominent role of OTUB1 in cancer, OTUB1 is a promising target in cancer therapy.
